# Effect of Co and Al Content on CrFeNiMo-System High Entropy Alloys Produced by Mechanical Alloying

**DOI:** 10.3390/ma18091936

**Published:** 2025-04-24

**Authors:** Laura Elena Geambazu, Ciprian Alexandru Manea, Ileana Mariana Mateș, Delia Pătroi, Gabriela Beatrice Sbârcea, Eugen Manta, Augustin Semenescu

**Affiliations:** 1 National Institute for R&D in Electrical Engineering ICPE-CA Bucharest, Splaiul Unirii 313, 030138 Bucharest, Romania; laura.geambazu@icpe-ca.ro (L.E.G.); delia.patroi@icpe-ca.ro (D.P.); gabriela.sbarcea@icpe-ca.ro (G.B.S.); eugen.manta@icpe-ca.ro (E.M.); 2 Faculty of Materials Science and Engineering, National University for Science & Technology Politehnica Bucharest, 313 Splaiul Independentei, 060042 Bucharest, Romania; augustin.semenescu@upb.ro; 3 Central Military Emergency University Hospital “Dr. Carol Davila”, Calea Plevnei 134, 010825 Bucharest, Romania; 4 Academy of Romanian Scientists, 3 Ilfov St., 050044 Bucharest, Romania

**Keywords:** mechanical alloying, high entropy alloys, solid-state processing, microstructural characterization

## Abstract

This study aims to investigate the Co content on a Co_x_CrFeNiMo (x = 0; 0.5) high entropy alloy (HEA) but also the effects of replacing the Co element with Al in terms of single-phase structure forming, processing behavior, and microstructural characteristics when being processed by mechanical alloying with a planetary ball mill. Recent HEA-related research aimed toward identifying the effect that certain alloying elements in different concentrations influence the microstructure and properties but also regulate their composition. HEAs present promising properties (e.g., corrosion and wear resistance) being applicable in domains that require protection against harsh environmental conditions, benefiting from the specific core effects of this type of material. To obtain a high alloying and homogenization degree, for this research, mechanical alloying was selected for processing the mixtures, with the aid of N-Heptane as a process control agent (PCA). The mixtures were monitored in terms of alloying degree evolution, elemental distribution, particle morphology, crystalline structure, and also technological characterization (packing ratio, free flow, and slope angle). The results indicated that a high degree of alloying was obtained after 30 h of solid-state processing, with notable crystallization of two major phases FCC and BCC identified confirming the HEA phase stability calculations.

## 1. Introduction

In the materials science field, high entropy alloys (HEAs) became a subject of very high interest due to various wish-for properties achieved by desirable elemental combinations. Overtime, studies [1,2,3,4,5,6,7,8] have proven the HEAs’ capabilities in aggressive environments where properties such as excellent resistance to oxidation, ductility and strength combination, and excellent resistance to corrosion, oxidation, and wear, which are mainly a result of the core features such as the cocktail effect [9], high configurational entropy [10], sluggish diffusion [11], and lattice distortion [12].

HEAs are known to have a much higher strength and a density comparable to steel [13], but they can be tailored by compositional design to meet specific criteria, being eligible to be utilized as improved performance coatings or parts in numerous applications and industries such as biomedicine, the power industry (catalysis and energy storage), soft magnetic materials, cutting tools, the aerospace industry, the geothermal environment, etc [14,15].

The elemental atomic proportion in HEAs has been studied over the years, observing the effect on the final properties. Mo, for example, could improve wear resistance, the stability of the passivation film, corrosion resistance, and lubrication [16,17,18,19] when added to the FeCoCrNi system HEA. The Mo content is also a key factor in the formation of hard intermetallic compounds [16,20]. Wang K. et al. [21] reported that Mo addition (0.3 mol) resulted in excellent brine corrosion resistance, but localized corrosion and pitting were identified when the Mo content was zero, indicating the impact of the elemental addition over Co_1.5_CrFeNi_1.5_Ta_0.1_Mo_x_ (x = 0, 0.1, 0.3, 0.5 mol).

Elements such as Co, Cr, Al, Cu [22,23], Ti, Nb, or Zr [24] were used in an attempt to combat the corrosion effects, where certain elements bring additional improvements. Cr is known for its protective properties by its passivating nature [25] and Cu for antibacterial and microbial-influenced corrosion [23]. Mn is known for its significant role in improving mechanical properties and twinning-induced plasticity [26,27].

Co is a widely used material for its corrosion resistance properties, but it is also known as an FCC phase stabilizer affecting the mechanical characteristics of multicomponent solid solutions [22,27,28,29]. Although the Co addition to HEAs theoretically improves the HEAs’ properties significantly in terms of phase stability and corrosion resistance for aggressive media such as geothermal and maritime fields [30,31], replacing or reducing its usage was classified as a top priority due to the global crisis of critical raw materials. The IRENA (International Renewable Energy Agency) and EERA (European Energy Research Alliance) expressed their concerns regarding the energy transition geopolitics and the role of research and innovation in the context of critical raw material supply for clean energy, where action is requested with a high priority, and where Co occupied the first position in the top of critical materials worldwide in 2023.

For this paper, Co content influence was studied in Co_x_CrFeNiMo, where x = 0; 0.5 but also how replacing the element with Al influences the single-phase structures, elemental distribution, particle shape and size, processing behavior, and material performance.

Al, such as Ni, is known for being enriched in the BCC phase [32], which indicates strength improvement for the final material according to the literature [33], making it an important addition to this study.

Mechanical alloying was selected as the processing route due to the different melting temperatures of the constituent elements, where the limitations of the liquid state processing might affect the alloying and homogeneity degree. The solid-state processing route is viable due to the HEAs’ nature of containing at least five elements in their composition, whereas casting and melting methods could lead to segregation or incomplete alloying [34].

## 2. Materials and Methods

For this research, CrFeNi-system advanced multicomponent materials were produced, where Al, Mo, and Co were added to improve the final materials’ properties in terms of wear and corrosion resistance. Three high entropy alloy (HEA) compositions were selected, namely CrFeNiMo (HEA-FREE), Co_0.5_CrFeNiMo (HEA-Co), and Al_0.5_CrFeNiMo (HEA-Al). In order to obtain the optimal experimental results, high-purity elemental metallic materials were mechanically alloyed, where Co, Cr, Fe, Ni, Mo, and Al were used as principal elements in the produced mixtures (HEAs). The commercial-grade materials are presented in Table 1.

In order to calculate the high entropy alloys’ phase stability [33], the valence electron concentration (VEC) was calculated using the following equation:

(1) $$VEC=\sum_{i=1}^{n}{VEC\;}_{i}\cdot c_{i}$$

where *n* is the number of elements, *VEC_i_* is the VEC of the *i* element, and *c_i_* is the *i*-element concentration.

To produce the proposed HEAs, mechanical alloying was selected due to the performances offered in terms of high alloying and homogenization degree by using a Retch PM400 (Retch, Haan, Germany) planetary ball mill equipped with 2 stainless steel vials and different sizes balls as the grinding media (dia. 10, 15, 20 mm). In order to avoid unnecessary welding of the material to the vial walls and balls, N-heptane 2 wt. % was used as a process control agent (PCA). Wet milling also brings benefits to the alloying process by reducing the necessary time and increasing the alloying efficiency. The metallic powders were manipulated in a glovebox with a controlled Ar atmosphere. Prior to the alloying process, the vials were purged with inert gas (Ar) in order to avoid supplementary oxidation, with the action being repeated after each collected sample. During the alloying process, several samples were collected in order to monitor the evolution of the alloying degree.

The XRD diffraction analysis D8-Discover diffractometer (Bruker, Rheinstetten, Germany) was utilized, equipped with a 1D LynxEye detector (Bruker AXS, Rheinstetten, Germany) and Cu primary radiation (λ = 1.540598 Å). The diffractograms were obtained in Braag–Bretano geometry with an angular increment of 0.04°, at a scanning speed of 1 s per step.

For the CrFeNiMo HEA crystallographic phase identification, the ICDD PDF 2 Release 2022 database was used, where assigned crystalline structures were found to be the following indexed files: PDF 01-086-8528, PDF 01-077-7591, PDF 03-065-7296, PDF 01-071-7597, PDF 01-077-7598, PDF 01-077-7959, PDF 03-065-7752, PDF 01-077-7973, PDF 01-071-9767, and PDF 01-077-6308.

The crystallographic phase identification for the Co_0.5_CeFeNiMo HEA was made with the same ICDD PDF 2 Release 2022 database, with the following indexed files: PDF 01-086-8528, PDF 01-077-7591, PDF 03-065-7296, PDF 01-071-7597, PDF 01-077-7598, PDF 01-077-7959, PDF 03-065-7752, PDF 01-077-7973, PDF 01-071-9767, PDF 01-077-6308, PDF 01-071-7326, PDF 01-082-3064, and PDF 01-082-3060.

The crystallographic phase identification for the Al_0.5_CeFeNiMo was made with the same ICDD PDF 2 Release 2022 database, with the following indexed files: PDF 01-086-8528, PDF 01-077-7591, PDF 03-065-7296, PDF 01-071-7597, PDF 01-077-7598, PDF 01-077-7959, PDF 03-065-7752, PDF 01-077-7973, PDF 01-071-9767, PDF 01-077-6308, PDF 01-071-7326, PDF 01-082-3064, PDF 01-082-3060, PDF 01-071-5710, PDF 01-081-9826, and PDF 01-073-8857.

The technological characterization of the HEAs produced was achieved by measuring free flow and tap density with a graduated cylinder, followed by the slope angle and flow rate using a calibrated Hall flow meter (Carney Funnel). In order to calculate the slope angle (α), the following equation was used.

(2) $$\alpha =arctg\frac{2h}{D}$$

The measurements and calculations were performed in order to determine the flowing capabilities of the metallic powders, characteristics necessary for spraying technique deposition methods ensuring smooth movement through the equipment tubes or pipes, but also a uniform packing density promotes defect-free final products when consolidating the powders through various methods.

## 3. Results and Discussions

The VEC calculations indicated the following:

$${VEC}_{CrFeNiMo}=7.5$$

$${VEC}_{{Co}_{0.5}CrFeNiMo}=7.67$$

$${VEC}_{{Al}_{0.5}CrFeNiMo}=7$$

According to the literature [33], the VEC predicts the structural properties based on the obtained values, where for the 6.8–8 interval, a mixture of FCC and BCC solid solution phase mixture was reported, with a balance between ductility and strength in the final HEAs. It was observed that even though the Co content was varied, along with being replaced with Al, the VEC value was in the same interval. It was also noted that for the Al_0.5_CrFeNiMo HEA, the obtained VEC value was seven indicating that the alloy could be more prone to have an abundance of BCC phases, suggesting a possible higher strength.

For the first alloy, it was decided to elaborate also a Co-free HEA (HEA-FREE) in order to observe the performances of the four principal elements in the equiatomic ratio material, reducing the critical material usage even further than the next HEAs. The milling parameters used were a 10:1 ball-to-powder ratio (BPR), and 320 RPM for 30 h in an Ar atmosphere. Samples were collected to determine the alloying degree at a 6 h period during the process and were XRD analyzed to observe the crystalline structure evolution. The samples were analyzed from the microstructural and chemical point of view and the results are presented.

**Figure 1:**
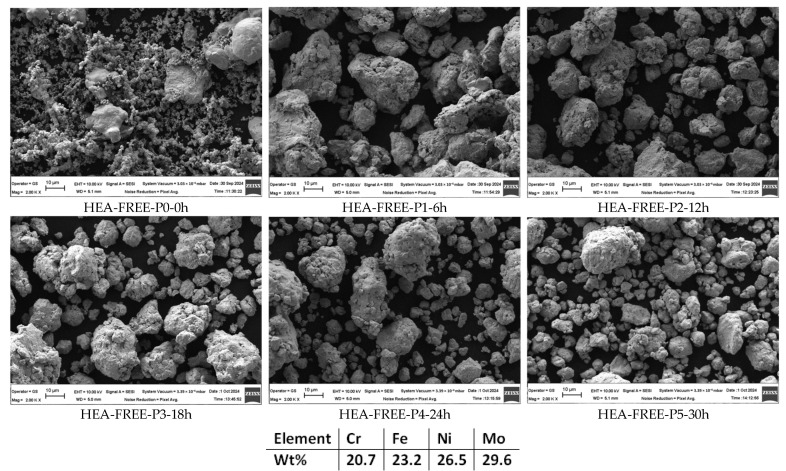
Alloying degree evolution and semi-quantitative EDS analysis results for HEA-FREE.

**Figure 2:**
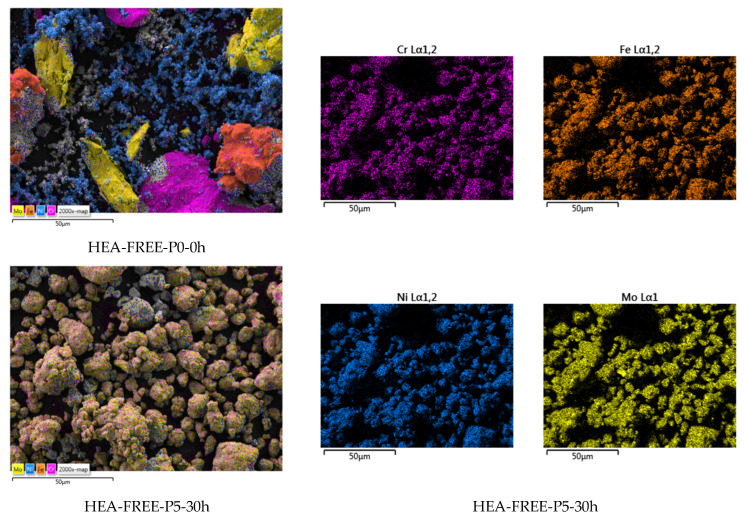
Elemental mapping analysis results for the HEA-FREE-P5-30h sample alloyed for 30 h compared to the HEA-FREE-P0-0h homogenized sample.

The alloying degree analysis results (Figure 1) present the evolution over a time span of 30 h for the CrFeNiMo HEA mixture. As it could be observed, the different metallic powder materials from the homogenized sample (HEA-FREE-P0-0h) could be identified by different shapes and sizes specific to each constituent element. Even from the early alloying stages, the shape of the particles started to change due to the welding and fracturing that is encountered during the mechanical alloying process. After 30 h, it was observed that no oxygen contamination occurred according to the EDS analysis; the particles have a polygonal shape and similar size concluding that the process was finished. The mapping analysis presented in Figure 2 indicates a high degree of alloying based on the elemental mapping, with a high degree of homogenization, and a clear distinction between the HEA-FREE-P0-0h and HEA-FREE-P5-30h samples.

**Figure 3:**
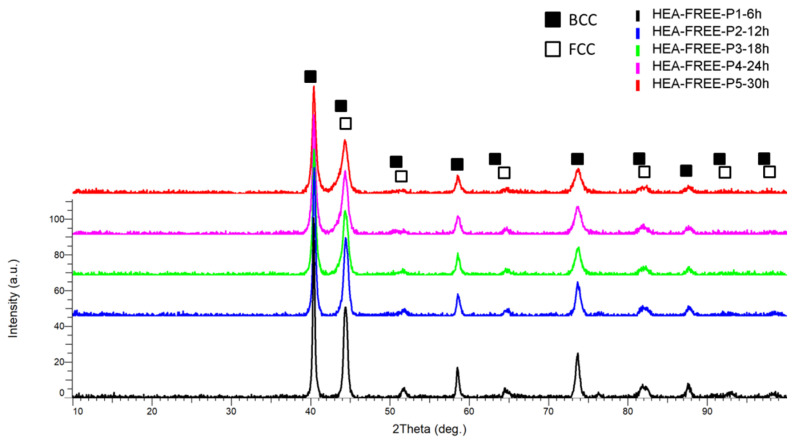
Comparative XRD patterns for the CrFeNiMo HEA produced by mechanical alloying.

The XRD comparative patterns are presented in Figure 3. It was observed that the highest peak intensity was identified at the 2θ angular position of 40.4° for all samples analyzed, and according to the similarities resulting from the indexed files, Cr, Fe, Ni, and Mo were present, indicating the alloying process, with a major FCC phase being present. The reflections for this peak presented an abundance of Mo, CrFe, CrNi, FeMo, FeNi, MoNi, and CrNi, confirming that the alloying process was produced.

The comparative results presented a decrease in the peaks’ intensity proportional to the alloying time, along with the peaks’ broadening, where it could be observed that for 30 h, the 2θ angular positions of 51.8°, 65°, 92.5°, and 98.5° are not present.

The second peak present at the 2θ angular position of 44.5° indicated the presence of Cr, Fe, Ni, and Mo with two major phases, namely FCC and BCC, where the BCC phase presented an abundance of Cr and Ni. The reflections for these phases were also identified at the 2θ angular position of 51.8°, 58.5°, 73.5°, 82°, 93°, and 98.5° with Cr, FeMo, FeNi, MoNi, and CrFe abundance.

**Figure 4:**
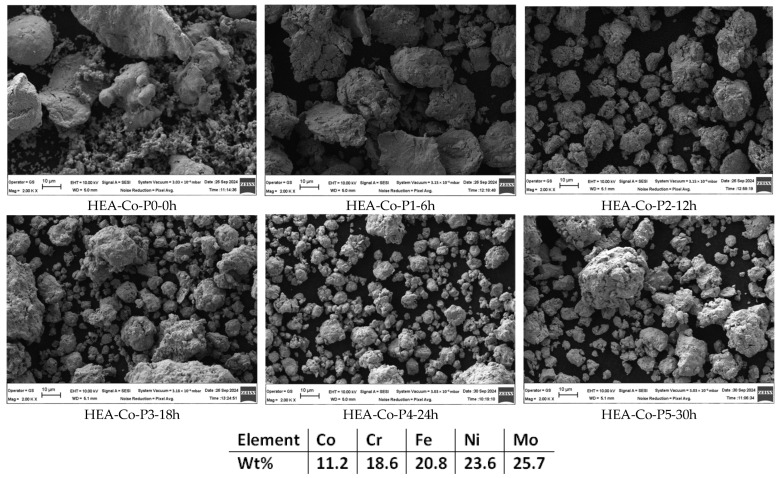
Alloying degree evolution and semi-quantitative EDS analysis results for HEA-Co.

For the Co_0.5_CrFeNiMo HEA (HEA-Co), the milling parameters were similar to the Co-free HEA, where the alloy was milled for 30 h at 320 RPM with a 10:1 BPR, with a mixture of stainless steel balls and in an Ar atmosphere. As PCA, N-heptane was used as previously described. As described in the literature [35], the intrinsic nature is affected by PCA in terms of crystal size refinement, solubility levels, and mechanical properties’ modifications due to the reaction that takes place between the agent and the processed material surface [36]. Samples were collected at 6, 12, 18, 24, and 30 h in order to observe the degree of alloying and homogenization. A sample of the initial homogenized mixture, namely HEA-Co-P0-0h, was also collected and analyzed to compare the results. The different particle shapes and sizes, presented in Figure 4, are present for this case as well. To avoid any unnecessary variation that could alter the results, the same metallic powders were used during the experimentation. As for the previous composition, a clear evolution degree was observed, and 30 h was sufficient for the mechanical alloying to be produced. There were no traces of oxygen identified and no other contaminants or impurities; the EDS analysis results therefore confirm the mixture composition.

**Figure 5:**
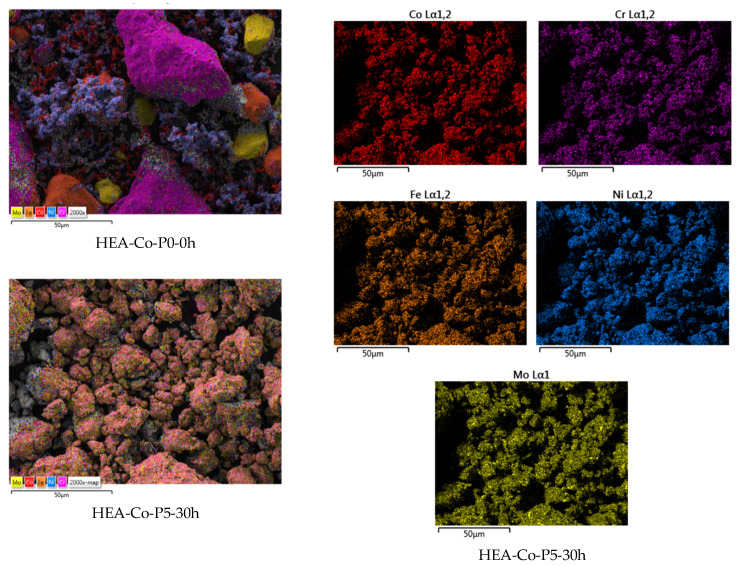
Elemental mapping analysis results for the HEA-Co-P5-30h sample alloyed for 30 h compared to the HEA-Co-P0-0h homogenized sample.

The elemental mapping analysis results presented in Figure 5 indicate a high alloying degree at intimate levels, and a very good elemental distribution, confirming that 30 h was sufficient for this experimentation stage, with a clear difference between the homogenized sample and the alloyed sample.

**Figure 6:**
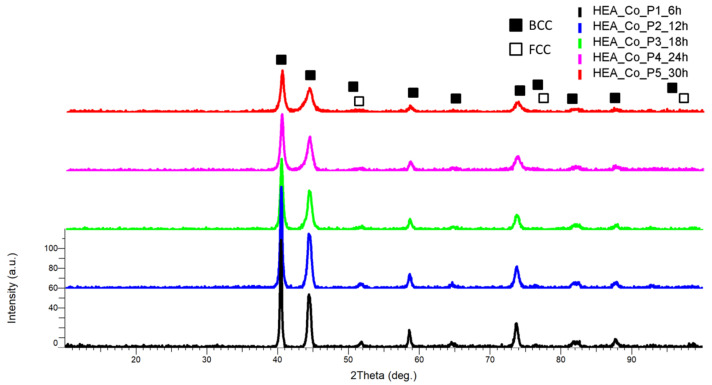
Comparative XRD analysis results for the Co_0.5_CrFeNiMo HEA produced by mechanical alloying.

Similar to CrFeNiMo HEA (HEA-FREE) (Figure 3), the comparative XRD results present a decrease in the peak intensity and peak broadening progressing with the alloying time (Figure 6), with the highest intensity peak identified at the 2θ angular position of 40.5° and the second peak intensity identified at the 2θ angular position of 44.5°. Two major phases were identified as BCC and FCC, a mixture confirmed also by the VEC calculations. The FCC phase, with CoNi abundance, was identified at the 2θ angular position of 52.2°, 76°, and 92.5°, while the BCC phase, abundant in CrNi, CrFe, FeMo and CoCr, was identified at the 2θ angular position of 40.5°, 44.5°, 52.2°, 58.5°, 64.8°, 74°, 76°, 82°, and 92.5° When compared to the obtained CrFeNiMo HEA, it was observed that the peaks shifted to the right, indicating a distortion or contraction in the crystalline structure. The observation indicates that dopant atoms of Co entered the crystalline structure. This result indicates that the Co was alloyed along with the other elements, confirming that the alloying process was produced.

The third composition selected was Al_0.5_CrFeNiMo HEA (HEA-Al). It was decided to add aluminum to the CrFeNi system due to its benefits such as improving machinability, corrosion resistance improvement, low density, and conductivity enhancement.

Aluminum is used in industry to improve alloys’ performance in numerous domains, one example being marine applications where the saline media induces a high degree of corrosion.

For the experimentation, the alloying parameters were decided based on the nature of the constituent chemical elements. Due to the aluminum addition, which facilitates the alloying process by its native properties, in this case, the mixture was alloyed at 270 RPM due to the ductility of aluminum and the greater tendency to weld versus break; a 10:1 BPR for 30 h with PCA and atmosphere parameters were used for the other two compositions.

**Figure 7:**
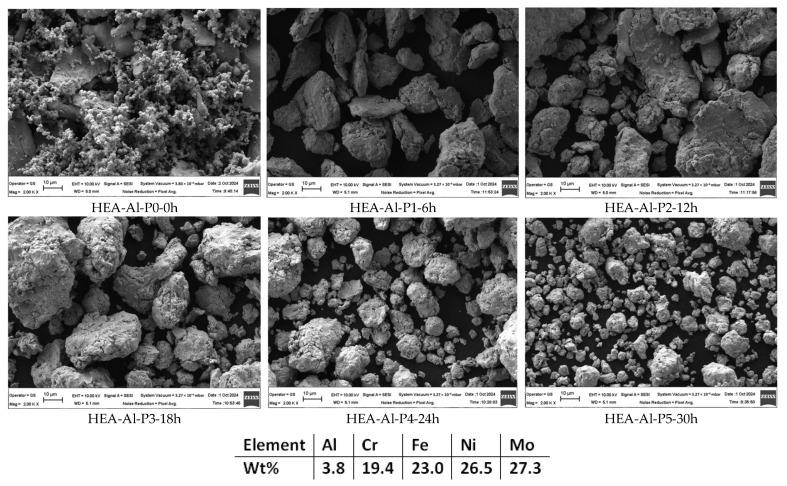
Alloying degree evolution and semi-quantitative EDS analysis results for HEA-Al.

As for the previous sets of samples, the mechanical alloying effect was observed from the first process stages in Figure 7, where from the various shapes and sizes, the particles were resized and reshaped into similar polygonal shapes, indicating that the parameters were optimal to obtain a homogeneous material. The EDS analysis results confirmed the composition and indicated that no traces of oxygen or impurities were detected. The results are favorable due to the Ar atmosphere used during ball milling processes, but also due to the wet milling aided with the N-heptane as PCA.

**Figure 8:**
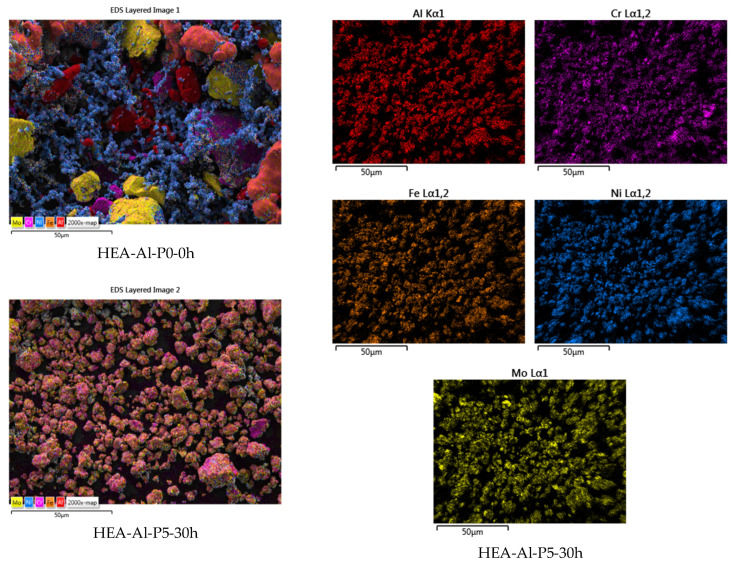
Elemental mapping analysis results for the HEA-Al-P5-30h sample alloyed for 30 h compared to the HEA-Al-P0-0h homogenized sample.

Although a lower RPM was used for the Al_0.5_CrFeNiMo HEA mixture, the elemental materials are evenly distributed throughout the entire analyzed surface according to the mapping analysis result presented in Figure 8.

**Figure 9:**
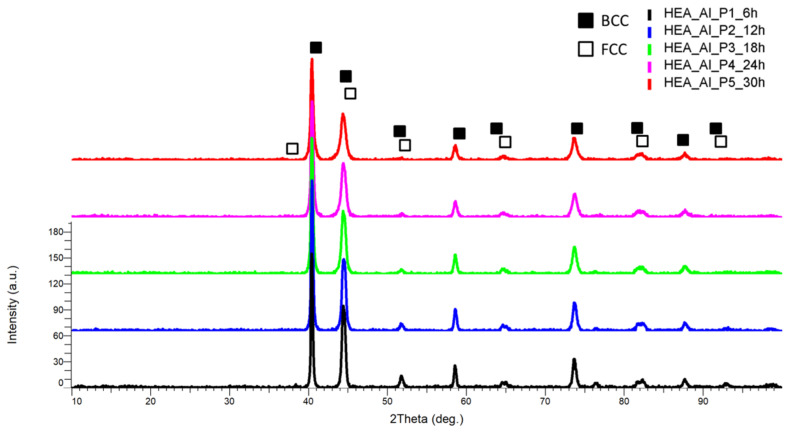
Comparative XRD analysis results for the Al_0.5_CrFeNiMo HEA produced by mechanical alloying.

For this HEA, it was observed that the highest peak intensity on the XRD graph was identified at the 2θ angular position of 40.5° (Figure 9), but here, there is a notable difference in intensity when compared to the other peaks. This results in a very good crystallization of the studied material sample. The FCC and BCC phase mixture is present, confirming the theoretical calculations, but an additional FCC single phase was identified at the 2θ angular position of 44.7°, 44.8°, 64.8°, 65°, 82°, and 82.5°, indicating an increased ductility in the final bulk material. The VEC calculation predictions were confirmed, and the BCC phase surpassed the FCC phase exhibiting the highest crystallinity as indicated by the peaks’ intensity. It could also be noted that the Al_0.5_CrFeNiMo HEA presented a greater degree of crystallinity and phase stability when compared to the CrNiFeMo and Co_0.5_CrFeNiMo HEAs.

It was observed that the oxidation of the metallic powders was under the detection limit of the analysis equipment for all produced HEAs due to the protective Ar atmosphere used during the glove box powder manipulation, but also during the mechanical alloying process.

According to the literature [35], mechanical alloying could be an efficient path to obtain superior properties in high entropy alloys due to the phase and microstructure modifications related to the process itself. It is also noted that elements such as Cr, V, Ti, and Al enhance the BCC phase formation [37], which is relevant to the studied case. Al influences mechanical but also physical properties in addition to phase formation control [38].

Another aspect was the lack of C content in the chemical analysis results. The literature presents that C contamination as traces is usually present when wet milling with PCA is used [39,40]. For the current case, 2 wt.% N-Heptane (containing approx. 84% C content) was used, but its presence could not be confirmed due to equipment limitations (detection limits).

In order to continue with the powder sintering, the technological characterization of the produced HEAs’ metallic powder is required. The compressibility can be predicted and characterized by tests such as free flow, tap density, flow rate, and slope angle.

The density obtained after consolidation depends on some characteristics of the powder, such as the following:

- Particle size;
- Particle size distribution;
- Particle shape.

The slope angle is the angle relative to the horizontal formed by the flowing powder and it is the most common method for statistically ensuring a good filling of the pressing die. The measurements and calculation results are presented in the following table, which includes all three HEAs produced by mechanical alloying.

As presented in Table 2, it was observed that the values obtained for the high entropy alloys are close to average when compared to the raw powder materials due to the mechanical alloying process. The packing ratio determined that the powder materials are suitable to be consolidated by spark plasma sintering, with the slope angle confirming the result. Although the slope angle values calculated for the studied HEA metallic powders are close to a satisfactory level, when a slope angle is higher than 45°, there is a high probability that it will not be suitable for the tablet compression process due to the poor die-filling degree. The addition of a flowing agent could be taken into consideration thus improving the flow property and reducing the risk of defects or uneven samples. For our case, the values are to be found in the interval of 35–41°, indicating that a flowing agent was considered in case of an unsatisfactory result.

From the results, it could be observed an improved flow for the Al_0.5_CrFeNiMo HEA when compared to the other proposed compositions in terms of slope angle degree but also free flow. The results are necessary indicators for further processing into consolidated materials, which could be used as bulk or complex geometry parts. In terms of the packing ratio, the values are similar between the three HEAs, indicating good capabilities of being pressed into tablets or targets for various processes. The results of this work will be further used as the basis for creating potential anticorrosive materials as parts or coatings due to their presented features.

## 4. Conclusions

From the alloying process, conclusions can be drawn regarding the mechanical alloying efficiency for all studied cases. It was noted that a time interval of 30 h was sufficient for this experimentation stage in order to achieve a very high alloying degree, with no contamination or oxidation.

The alloys presented a high homogeneity degree and high alloying degree aided by the use of N-heptane as PCA. Although the C content was taken into consideration for the final results, no traces were detected by the equipment used for the chemical analysis.

The phase stability calculations indicated the possibility of FCC and BCC phases and were confirmed by the XRD analysis results with an improved crystallinity and phase stability for the Al_0.5_CrFeNiMo HEA, where the BCC phase surpassed other phases indicating a strengthening potential of the final material.

Metallic powder technical characterization indicated that the materials are suitable for further processing into a bulk state with methods such as spark plasma sintering or classic consolidation methods having a mean packing ratio of approx. 75% for all three studied HEAs.

Future work will be concentrated on obtaining bulk materials to be further used as electrodes for the electro-spark deposition method, with the end goal of producing corrosion-resistant coatings for aggressive environments.

## Figures and Tables

**Table 1:** Elemental metallic powders used for the multicomponent materials’ development.

Elemental Material	Particle Size (µm)	Purity (%)
Cobalt (Co)	37	99.5
Iron (Fe)	60	>99
Chromium (Cr)	<45	>99
Nickel (Ni)	<50	99.7
Aluminum (Al)	15	99.5
Molybdenum (Mo)	53–90	>99

**Table 2:** Elemental material and HEA metallic powder characterization.

Metallic Powder	Free Flow Density (g/cm^3^)	Tap Density (g/cm^3^)	Packing Ratio (%)	Free Flow (g/s)	Slope Angle (°)
Al	1.21	1.47	82.3	1.09	17.74
Co	2.08	2.6	80	0.57	34.21
Cr	2.45	3.2	76.6	1.2	31.79
Fe	3.12	4.05	77	0.85	9.64
Ni	4.54	5.1	89	12.19	17.74
Mo	4.16	4.63	89.8	9.61	21.8
CrFeNiMo	3.45	4.44	77.7	2.47	39.72
Co_0.5_CrFeNiMo	3.51	4.65	75.5	2.56	40.91
Al_0.5_CrFeNiMo	3.28	4.35	75.4	4.53	35.29

## Data Availability

The original contributions presented in this study are included in the article. Further inquiries can be directed to the corresponding authors.
